# Some Eminent Misers

**Published:** 1892-12

**Authors:** 


### SOME EMINENT MISERS.

Occasionally men have become miserly from good motives ; as did an Italian physician, who denied himself the common necessaries of life, and when he died was mourned by none until his will was read, when it was learned that he left his entire fortune to be expended in bringing water from the mountain to his native village. So, also, when Bethlehem hospital was built an East End miser gave a donation of £100. When the collector called for the amount, he was found scolding a servant for throwing away a match which hand not been burned at both ends. Misers are not confined to one class of the community, but have been, at least, as common to the higher ranks as to the lower. John Churchill, first duke of Marlborough, was the greatest soldier in Europe. Yet, when he was an old man, in order to save sixpence from carriage hire, he would walk from the public rooms in Bath to his hotel in all kinds of weather. He died worth £1,000,000, which reverted to his bitterest enemy, his grandson, Lord Trevors.

Sir Harvey Elwes, of Stoke, in Suffolk, next to hoarding money, found his principal pleasure in netting partridges. He and his household, consisting of one man and two maids, lived upon these. In cold or wet weather Sir Harvey would walk up and down his hall to save fire. His clothes cost him nothing, for he ransacked old chests and wardrobes and wore those of his ancestors. When he died the only tear shed was by his servant, to whom he left a farm, value, £50 per annum. The whole of his property was left to his nephew, John Maggott, who thus inherited real and personal estate valued at £250,000, on condition that he would assume the name and arms of Elwes. Of this man, who is better known as John Elwes the miser, the following story is told: His nephew, Colonel Timms, visited him at Marcham, and, after retiring to rest, found himself wet through. Finding that the rain was dripping through the ceiling, he moved the bed. He had not lain long before the same inconvenience again occurred. Again he arose, and again the rain came down. After pushing the bed quite around the room, he found a corner where the ceiling was better secured, and slept until morning. When he met his uncle at breakfast he told him what had happened. "Aye, aye," said Mr. Elwes, "I don't mind it myself, but to those who do, that's a nice corner in the rain." Mr. and Miss Dancer were reputed the most notorious misers of the eighteenth century. The manner in which this couple were found, after death, to have disposed of their wealth was even more strange than could have been their methods of acquiring it. The total value was £20,000 which was thus disposed of: Two thousand five hundred pounds was found under a dunghill; £500 in an old coat nailed to the manger in the stable; £600 in notes were hidden away in an old teapot; the chimney yielded £2,000, stowed in nineteen separate crevices. Several jugs filled with coin were secreted in the stable loft.

Rev. Mr. Jones, of Blewbury, with a nest egg of £200 and a stipend amounting to £50 per annum, left at death the sum of £10,000. He had been rector of his parish for forty years, and during all that time only one person had been known to sit at his festal table. No fire was ever lighted in his house, nor was a servant kept. In winter he would visit his parishioners, to keep himself from starving of cold, rather than light a fire at the rectory. As like affects like, so it is with misers; and gold will go where gold is. This is strikingly illustrated by the act of a celebrated Greek, one Dichoeus Dichoenus, a descendant of the Byzantine emperors. This man, by the exercise of extreme niggardliness, managed to amass the sum of £10,000—an immense fortune in those days. Then came the question, to whom should he leave it. One day a distant relative sent him a letter written upon a square inch of paper; this was sufficient. In the fitness of things the parsimonious correspondent became the miser's heir.

It has sometimes happened that persons little deserving, and even rulers, have reaped the harvests which misers have painfully sown. The life of Vandille is a proof of this. The man lived upon bread and milk, with the addition of a small glass of sour wine on Saturdays. At his death he left £800,000 to the king of France. Audley, the commonwealth miser, saved £400,000, all of which reverted to the government. A merchant died at Ispahan in the earlier part of this century, who had for many years denied himself and his son every support except a crust of coarse bread. On a certain occasion he was overtempted to buy a piece of cheese, but, reproaching himself with extravagance, he put the cheese into a bottle and contented himself, and obliged the boy to do the same, with rubbing the crust against the bottle, enjoying the cheese in imagination. One day, returning home later than usual, the merchant found his son eating his crust, which he constantly rubbed against the door. "What are you about, you fool?" was his exclamation. "It is dinner time, father. You have the key, so, as I could not open the door, I was rubbing my bread against it, as I could not get to the bottle." "Cannot you go without cheese one day, you luxurious little rascal? You'll never be rich." And the angry miser kicked the poor boy for not having been able to deny himself the ideal gratification.—*Cassell's Saturday Journal.*

